# Supplementary material for: Identification of photoperiod-induced specific miRNAs in the adrenal glands of Sunite sheep (Ovis aries)
Source: Front Vet Sci. 2022 Jul 22;9:888207. doi: 10.3389/fvets.2022.888207 (PMC9354845; doi:10.3389/fvets.2022.888207)
Supplement: Supplementary file 1 [file Data_Sheet_1.ZIP › Supplementary file/miRDeep2 file.zip!/miRDeep2 file/Novel_Structure/SP42A1/Novel_222.pdf]

Provisional ID	:	NC_019467.2_13209
Score total	:	1.6
Score for star read(s)	:	-1.3
Score for read counts	:	0
Score for mfe	:	1.3
Score for randfold	:	1.6
Score for cons. seed	:	
Total read count	:	1570
Mature read count	:	1570
Loop read count	:	0
Star read count	:	0

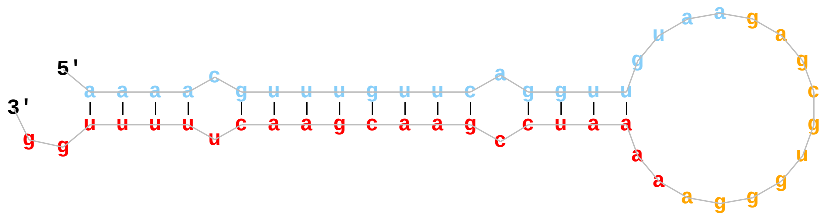

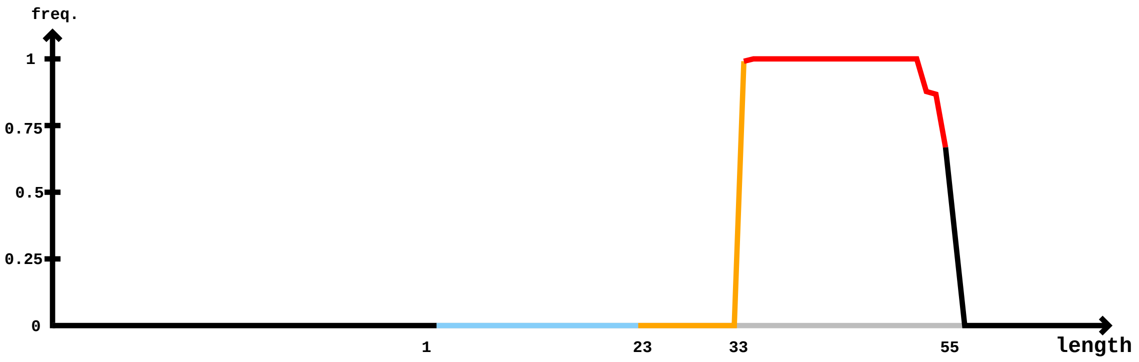


```
                                      Star                          Mature
5'- uggguucuguccuagguaaugguaguacuggguuggccaaaacguuuguucagguuguaagagcgugggaaaaauccgaacgaacuuuuuggccaagucaauaauuug   -3'    exp
    ..(((.(((.((........))))).)))((.((((((((((.((((((((.((((................)))).)))))))).)))))))))).)).........    reads      mm         sample
    ......................................................................aaaauccgaacgaacuuuu...................    192        0          seq
    ......................................................................aaaauccgaacAaacuuuuu..................    2          1          seq
    ......................................................................aaaauccgaacgaacuuuuug.................    313        0          seq
    ......................................................................aaaauccgaacgaacuuuuugg................    1049       0          seq
    .......................................................................aaauccgaacgaacuuuuu..................    14         0          seq
```
